# Interval cancer in the Córdoba Breast Tomosynthesis Screening Trial (CBTST): comparison of digital breast tomosynthesis plus digital mammography to digital mammography alone

**DOI:** 10.1007/s00330-023-10546-x

**Published:** 2024-01-04

**Authors:** Cristina Pulido-Carmona, Sara Romero-Martín, José Luis Raya-Povedano, María Cara-García, Pilar Font-Ugalde, Esperanza Elías-Cabot, Margarita Pedrosa-Garriguet, Marina Álvarez-Benito

**Affiliations:** 1 Maimónides Biomedical Research Institute of Córdoba (IMIBIC), Córdoba, Spain; 2 Breast Cancer Unit, Department of Diagnostic Radiology, Reina Sofía University Hospital, Avenida Menéndez Pidal s/n, 14004 Córdoba, Spain; 3 University of Córdoba, Córdoba, Spain; 4 Rheumatology Department, Reina Sofía University Hospital, Avenida Menéndez Pidal s/n, 14004 Córdoba, Spain

**Keywords:** Breast neoplasms, Digital breast tomosynthesis, Mammography, Mass screening

## Abstract

**Purpose:**

This work aims to compare the interval cancer rate and interval cancer characteristics between women screened with digital breast tomosynthesis (DBT) + digital mammography (DM) and those screened with DM alone.

**Methods:**

The interval cancer rate and interval cancer characteristics of the study population included in the Córdoba Breast Tomosynthesis Screening Trial (CBTST) were compared to a contemporary control population screened with DM. The tumour characteristics of screen-detected and interval cancers were also compared. Contingency tables were used to compare interval cancer rates. The chi-square test and Fisher’s exact test were used to compare the qualitative characteristics of the cancers whereas Student’s *t* test and the Mann–Whitney *U* test were used to analyse quantitative features.

**Results:**

A total of 16,068 screening exams with DBT + DM were conducted within the CBTST (mean age 57.59 ± 5.9 [SD]) between January 2015 and December 2016 (study population). In parallel, 23,787 women (mean age 58.89 ± 5.9 standard deviation [SD]) were screened with DM (control population). The interval cancer rate was lower in the study population than in the control population (15 [0.93‰; 95% confidence interval (CI): 0.73, 1.14] vs 43 [1.8‰; 95% CI: 1.58, 2.04] respectively; *p* = 0.045). The difference in rate was more marked in women with dense breasts (0.95‰ in the study population vs 3.17‰ in the control population; *p* = 0.031). Interval cancers were smaller in the study population than in the control population (*p* = 0.031).

**Conclusions:**

The interval cancer rate was lower in women screened with DBT + DM compared to those screened with DM alone. These differences were more pronounced in women with dense breasts.

**Clinical relevance statement:**

Women screened using tomosynthesis and digital mammography had a lower rate of interval cancer than women screened with digital mammography, with the greatest difference in the interval cancer rate observed in women with dense breasts.

**Key Points:**

• *The interval cancer rate was lower in the study population (digital breast tomosynthesis [DBT]* + *digital mammography [DM]) than in the control population (DM).*

• *The difference in interval cancer rates was more pronounced in women with dense breasts*.

• *Interval cancers were smaller in the study population (DBT* + *DM) than in the control population (DM)*.

## Introduction

The widespread use of breast cancer screening programmes has reduced the mortality associated with this disease as well as the aggressiveness of treatment, given that cancer can be diagnosed in earlier stages [1].

At present, digital mammography (DM) is the most accepted technique for breast cancer screening. Nonetheless, this technique has limitations, including a sensitivity of 70–90% [2], which can even be as low as 47.8–64.4% in women with dense breasts [3], and a specificity with a false positive rate of between 8 and 14% [4]. Digital breast tomosynthesis (DBT) is a novel technique that creates a pseudo-three-dimensional view of the breast. Various publications on DBT have shown that it improves screening sensitivity, with variable results on recall [5, 6].

Interval cancer is one of the drawbacks of screening programmes. Its incidence is determined by the natural history of the disease and the frequency and sensitivity of screening tests; the goal is to achieve an interval cancer rate that is as low as possible. A high proportion of interval cancers will reduce the effectiveness of a screening programme and its impact on reducing mortality [7–12].

The increased detection rate achieved with DBT seems to suggest that this technique may be associated with a decreased rate of interval cancers. Several publications have not confirmed this hypothesis [13–20]. However, a recent publication which included women from the “Malmö Breast Tomosynthesis Screening Trial” did indeed show a lower rate of interval cancers in women screened with DBT compared to those screened with DM [21].

Given the conflicting results in the literature, more data are needed to better clarify the effect of DBT on the interval cancer rate. In the Córdoba Breast Tomosynthesis Screening Trial (CBTST), conducted by this research group, favourable results have already been obtained on DBT in cancer detection and recalls [22]. In this study, the impact of DBT on interval cancer in the population included in the CBTST (study population) was assessed compared to women screened with DM only in the same round (control population), comparing differences in cancer rates and characteristics. Furthermore, differences between screen-detected and interval cancers were also evaluated.

## Methods

This study included retrospectively collected screening examinations. The use of these data was approved by the hospital’s institutional review board and the requirement for informed consent was waived.

### Study and control population

The population breast cancer screening programme in Córdoba consists of bilateral mammography with double projection on a biennial basis for women aged 50 to 69 years. Double reading is performed without consensus or arbitration of these studies (women are recalled if any reader decides to recall).

From January 2015 to December 2016, 18,665 women were invited to participate in the prospective CBTST study [22], whose main objective was to compare DBT to DM in terms of detection and recall rates. DBT and DM (Selenia Dimension device, Hologic) with double projection of each breast for each modality were simultaneously conducted on the 16,067 women who agreed to participate in the study (study population). Given that bilateral cancer was detected in one patient, the final results are on 16,068 screening exams.

The control population comprised 23,787 women who were studied in the same period with two different DM devices (Senographe Essential devices, GE or Lorad Selenia devices, Hologic). DM with double projection of each breast was evaluated in these subjects.

**Fig. 1:**
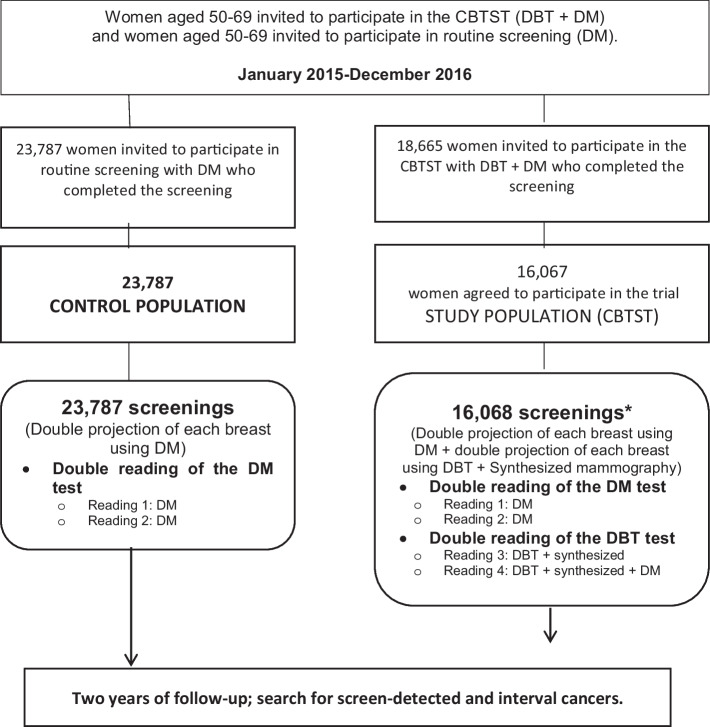
Flowchart of data selection and collection in the study. In total, 39,855 women were included: 23,787 in the control population and 16,067 in the study population. *One woman in the study population had bilateral cancer; thus, 16,068 screening exams were ultimately included. CBTST, Córdoba Breast Tomosynthesis Screening Trial; DBT, digital breast tomosynthesis; DM, digital mammography

The reading of the studies from both populations was conducted by five radiologists (including J.L.R.P., S.R.M., M.C.G., M.P.G.), who have between 3 and 15 years of experience in breast imaging and screening mammography reading. In the control population, double reading was performed for each study. In the study population, four readings were performed: double reading of the results from each imaging technique (DM and DBT). All readings were blinded and independent, without consensus or arbitration. No radiologist performed more than one reading for each woman. Women were recalled if any reader decided to recall them. Further testing was conducted if needed (Fig. 1).

### Screen-detected and interval cancer definition

Screen-detected cancer was defined as breast cancer identified by a screening test in women who were part of the target population (7–9). Interval cancer was defined as primary breast cancer diagnosed in women who had a previous negative screening test for malignancy in the period before the next invitation for screening within the programme or within the period of time equal to the screening interval (2 years) in the event a woman aged out of the programme (7–9).

After a follow-up period of at least 2 years (interval used in the screening programme), a thorough search was conducted using different sources to identify all screen-detected cancers and all interval cancers in the following populations: Official Tumour Registry of Córdoba, Spain; data generated by the Early Detection Programme in Andalusia, Spain; the Spanish Minimum Basic Data Set (MBDS) (mandatory medical record database for hospitals in Spain’s National Health System); the Clinical Health History and the database of the centre’s Breast Unit.

### Variables, indicators, and comparisons

The variables collected included screening date, age, modality used (DM or DBT + DM), breast density according to the Breast Imaging Reporting and Data System (BI-RADS) classification, number of recalls, and diagnosis of screen-detected cancer or interval cancer. In women diagnosed with cancer, the date of diagnosis, radiological finding, stage, histopathological characteristics, and treatment were also collected. In patients diagnosed with interval cancer, the reason for the diagnosis (onset of symptoms or imaging test performed outside the screening programme before the next invitation for screening within the programme) and the time from the negative screening exam to the cancer diagnosis were also recorded. Breast density was defined based on the DM studies in both the study population and the control population. If the two readings did not coincide, the one with highest density was chosen.

The rate of interval cancer (number of interval cancers per 1000 women screened) was calculated independently for the two populations (study and control) and stratified according to age group (under and over 60 years), breast density (A/B vs C/D), and time between interval cancer diagnosis and previous negative screening (< 12 months vs ≥12 months). The results of the study and control populations were compared.

The sensitivity of the screening programme (number of screen-detected cancers/number of screen-detected cancers + number of interval cancers) was also calculated independently for both populations (study and control) and stratified by age group (under and over 60 years). The results of both populations were compared. Moreover, the rate differences (RD) between both populations in each case were calculated.

Histopathological characteristics, stage, and treatment of the interval cancers were compared between study and control populations. Those variables were also compared between interval cancers and screen-detected cancers when both populations were analysed together.

### Statistical analysis

The descriptive study was conducted by calculating the arithmetic mean and standard deviation for quantitative variables and absolute and relative frequencies (%) for qualitative variables. For risk estimation, the odds ratio (OR) was calculated. Confidence intervals were calculated for a 95% confidence level.

Bivariate analysis was performed using the chi-square test and Fisher’s exact test for qualitative data and Student’s *t*-test and the Mann–Whitney *U* test for quantitative data if the criteria of normality were not met.

Two-tailed statistical tests were used. The *p* values were adjusted for all comparisons using the Benjamini–Hochberg method and were considered significant when *p* < 0.05.

The data were stored, processed, and analysed using the SPSS Statistics programme version 25.0 (IBM, 2017).

## Results

A total of 16,068 screening exams were performed on the study population (mean age 57.59 ± 5.9 years). Ninety-seven women had screen-detected cancer (one of them bilateral, 98 cancers detected in total, cancer detection rate (CDR) 6.09‰) and 15 had interval cancers (interval cancer rate (ICR) 0.93‰) (Table 1).

**Table 1:** General characteristics of the subjects included in the study

	Study population (CBTST)	Control population	Total	*p* value	Adjusted *p* value
Sample	16,068 (40.3)	23,787 (59.7)	39,855 (100)	-	-
Age at screening (years) ^	57.59 ± 5.9	58.89 ± 5.9	58.36 ± 5.9	< 0.001	< 0.001
Age groups (years)	6204 (38.6)	6812 (28.6)	13,016 (32.7)	< 0.001	< 0.001
• 50–54*	3823 (23.8)	6455 (27.1)	10,278 (25.8)		
• 55–59	3354 (20.9)	5326 (22.4)	8690 (21.8)		
• 60–64	2687 (16.7)	5184 (21.8)	7871 (19.7)		
• 65–70**					
Density”				< 0.001	< 0.001
• Categories A + B	11,861 (73.8)	14,702 (61.8)	26,563 (66.64)		
• Categories C + D	4207 (26.2)	8950 (37.62)	13,157 (33.01)		
Original outcomes					
• Negative	14,872 (92.6)	22,416 (94.2)	37,288 (93.6)	< 0.001	< 0.001
• False positive	1098 (6.83)	1225 (5.15)	2323 (5.82)	0.034	0.056
• Screen-detected cancer [*N* (CDR)]	98 (6.09/1000)	146 (6.13/1000)	244 (6.12/1000)	0.961	0.961
• Interval cancer [*N* (ICR)]	15 (0.93/1000)	43 (1.8/1000)	58 (1.45/1000)	0.025	0.045
• Interval cancer (%)^ɣ^	15 (11.7)	43 (18.5)	58 (16.1)	0.092	0.96

*CBTST*, Córdoba Breast Tomosynthesis Screening Trial; *CDR*, cancer detection rate; *ICR*, interval cancer rate; *N*, number

Unless otherwise indicated, data are number of studies (percentage)

^Data are shown as mean ± standard deviation

^*^In this range, patients 49 years of age who were called for screening are included (43, included in the DM population)

^**^In this range, patients aged 71 years are included (1, included in the DM population)

**”**Breast Imaging Reporting and Data System (BI-RADS) classification

^ɣ^Percentage representing interval cancers out of total cancers in the population

A total of 23,787 screening exams were performed on the contemporary control population (mean age 58.89 ± 5.9 years). One hundred and forty-six women had screen-detected cancers (CDR 6.13‰) and 43 had interval cancers (ICR 1.8‰).

**Fig. 2:**
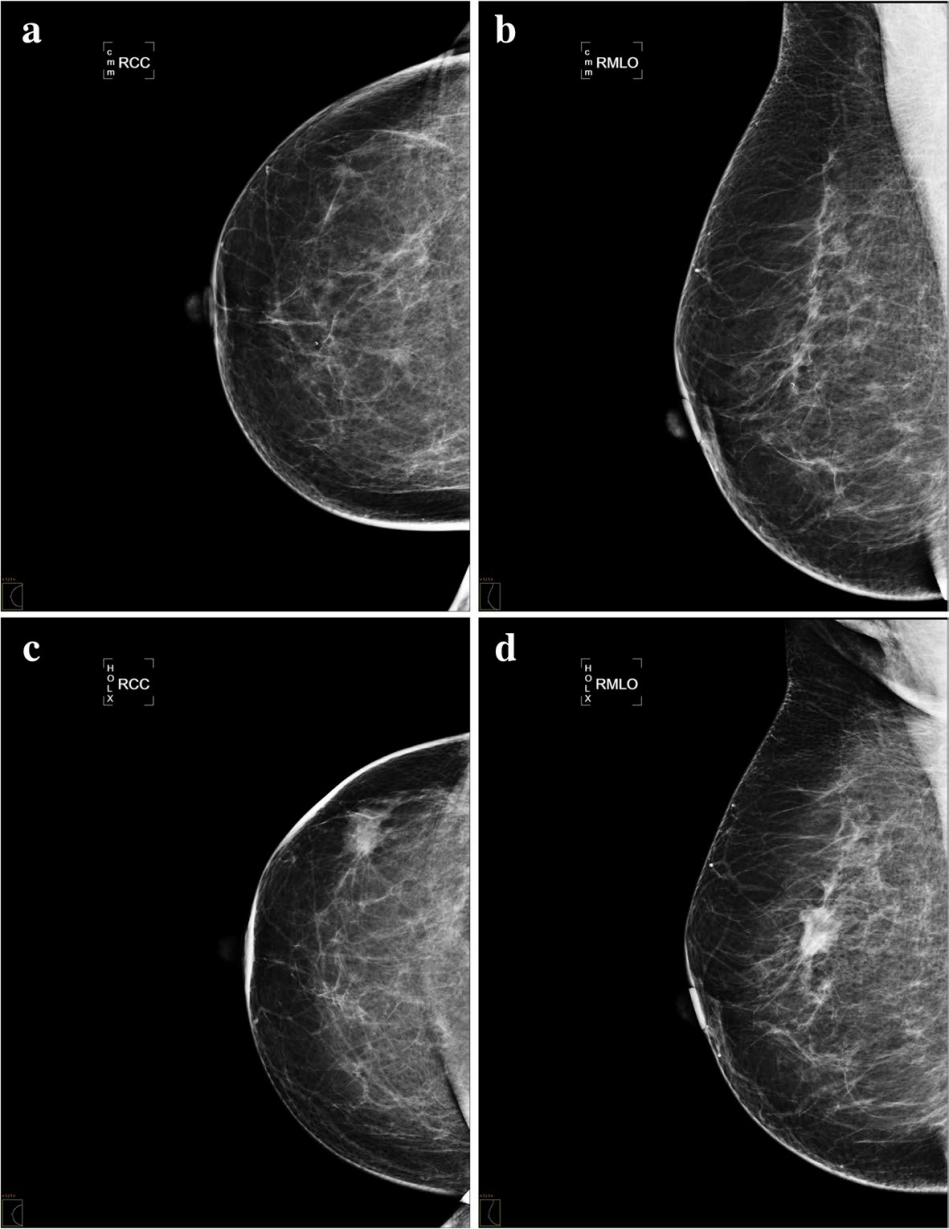
DM interval carcinoma. A 59-year-old woman with a palpable lump on the upper outer quadrant of the right breast 20 months after the last screening mammography, which was interpreted as normal. Craniocaudal (**a**) and right mediolateral oblique (**b**) projections of the screening mammography in which no suspicious findings were identified. Craniocaudal (**c**) and right mediolateral oblique (**d**) projections of diagnostic mammography, in which a spiculated mass (arrow) is seen in the symptomatic area. A percutaneous biopsy revealed a luminal B grade 2 invasive lobular carcinoma

**Fig. 3:**
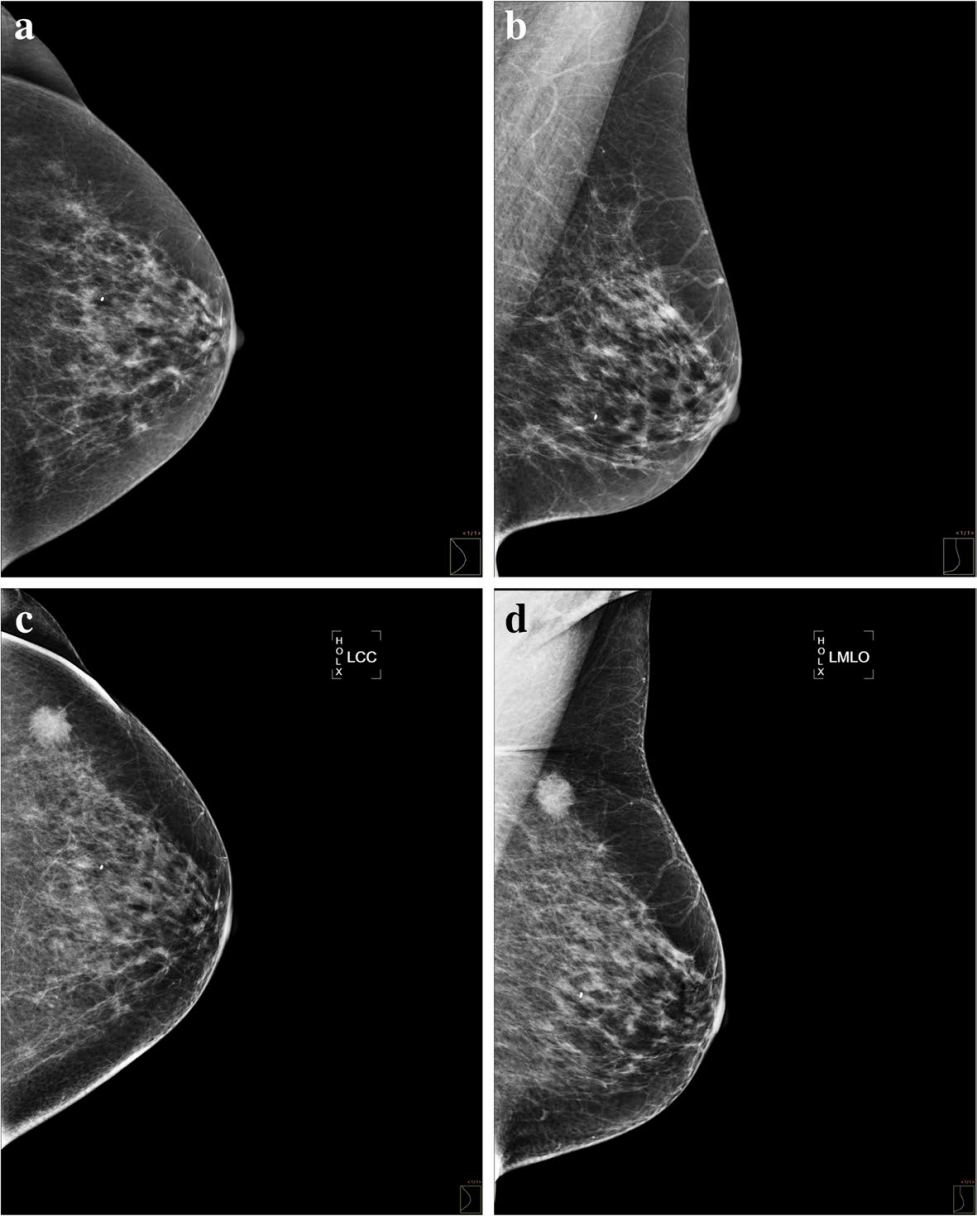
DM interval carcinoma. A 57-year-old woman with a palpable lump on the upper outer quadrant of the left breast 15 months after the last screening mammography, which was interpreted as normal. Craniocaudal (**a**) and left mediolateral oblique (**b**) projections of screening mammography without notable findings. Craniocaudal (**c**) and left mediolateral oblique (**d**) projections of diagnostic mammography, in which a spiculated mass (arrow) is identified in the symptomatic area. A triple-negative invasive ductal carcinoma with axillary involvement was confirmed

**Fig. 4:**
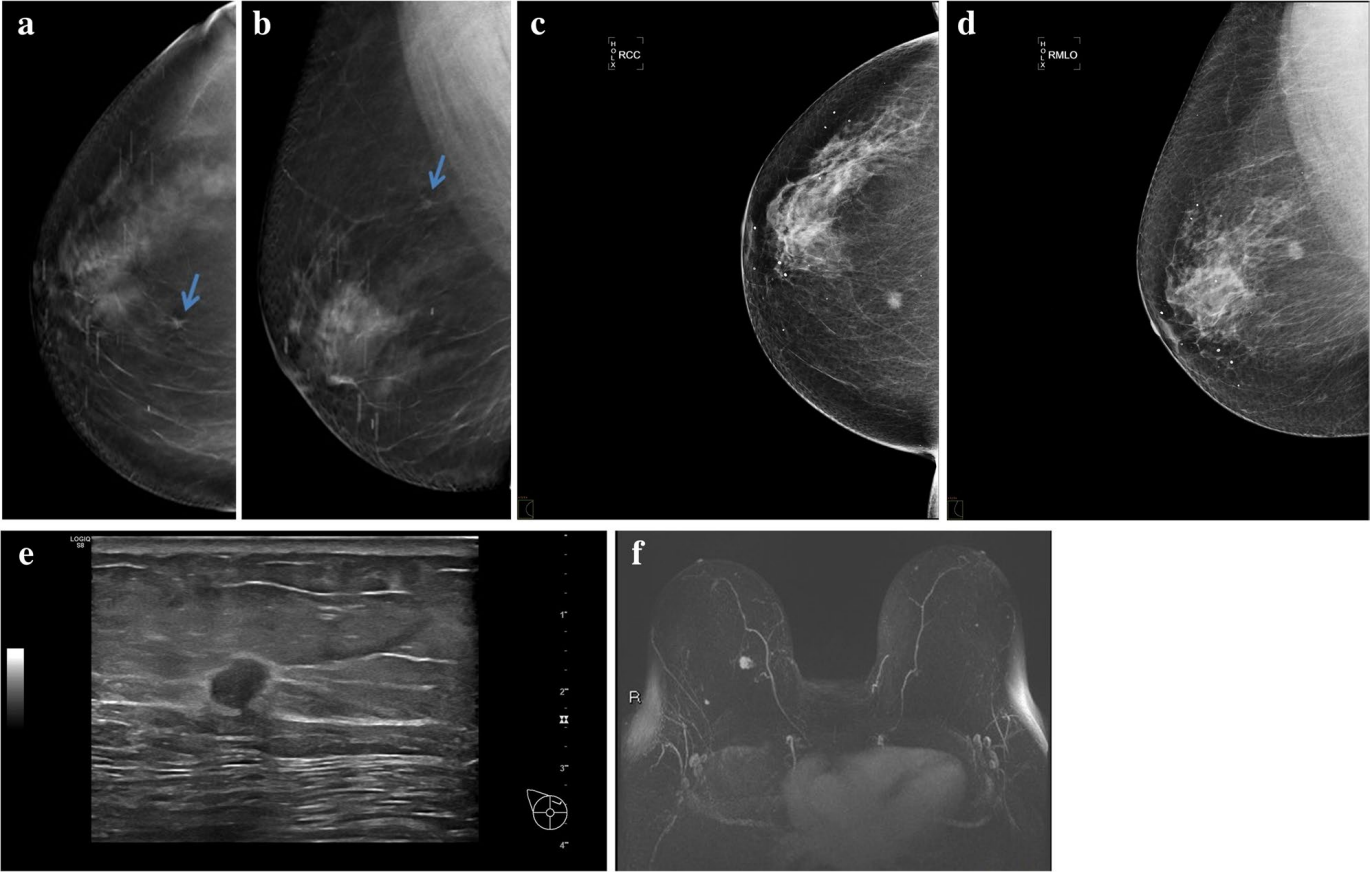
DBT interval carcinoma. A 60-year-old asymptomatic patient who underwent a repeat mammographic study 20 months after the screening study interpreted as normal due to the diagnosis of breast cancer in a first-degree relative. Craniocaudal (**a**) and mediolateral oblique (**b**) projections, respectively, of the tomosynthesis of the right breast performed in the screening program, in which a small spiculated mass is identified in the upper inner quadrant of the right breast (arrow) that was not noticed at the time. Craniocaudal (**c**) and mediolateral oblique (**d**) projections, respectively, of the diagnostic mammogram showing that the mass had increased in size. **e** Breast ultrasound, in which the mass is visible. **f** Breast MRI. MIP T1 reconstruction with IV contrast and fat suppression in which it is verified that the lesion is unique (arrow). Pathological result: luminal A grade 2 invasive ductal carcinoma

Most women with interval cancers (53/58; 91.37%) had breast symptoms (palpable lump, discharge, mastalgia, or other). One patient (1.81%) presented with vertebral metastases of unknown origin. In two patients (3.44%), interval carcinoma was diagnosed from an imaging test performed outside the screening programme before the invitation to the next round due to the diagnosis of breast cancer in a relative. In two patients (3.44%), the reason for the interval cancer diagnosis was unknown (Figs. 2, 3, and 4).

### Interval cancer rate

The interval cancer rate was significantly lower in the study population than in the control population (0.93‰; 95% CI: 0.73, 1.14 vs 1.8‰; 95% CI: 1.58, 2.04 respectively; *p* = 0.045). The OR was 0.51 (95% CI: 0.28, 0.93), which indicates that the probability of being diagnosed with an interval cancer was 49% lower in the women screened with DBT + DM (Table 2).

**Table 2:** Interval cancer rate and sensitivity. Differences between study and control population

	Study population (CBTST)	Control population	Rate difference (95% CI)	*p* value (X2)	Adjusted *p* value (X2)	OR (95% CI) *p* value	Adjusted *p* value (OR)
Number and interval cancer rate							
Global	15 0.93‰ (0.73, 1.14)	43 1.8‰ (1.58, 2.04)	− 0.87 (− 1.59, − 0.16)	0.025	0.045	0.51 (0.28, 0.93) *p* = 0.028	*p* = 0.047
Number and interval cancer rate according to age groups							
< 60 years	10 0.99‰ (0.73, 1.2)	25 1.88‰ (1.57, 2.12)	− 0.89 (− 1.84, 0.07)	0.084	0.160	0.52 (0.25, 1.1) *p* = 0.089	*p* = 0.144
≥ 60 years	5 0.82‰ (0.52, 1.14)	18 1.71‰ (1.37, 2.05)	− 0.88 (− 1.96, 0.19)	0.142	0.212	0.48 (0.18, 1.3) *p* = 0.151	*p* = 0.345
Number and interval cancer rate according to breast density							
Category A and B	11 (1 A; 10 B) 0.92‰ (0.69, 1.16)	15 (2 A; 13 B) 1.02‰ (0.80, 1.24)	− 0.09 (− 0.85, 0.66)	0.81	0.857	0.9 (0.41, 1.98) *p* = 0.81	*p* = 0.846
Category C and D	4 (3 C; 1 D) 0.95‰ (0.55, 1.35)	28 (25 C; 3 D) 3.17‰ (2.64, 3.62)	− 2.18 (− 3.66, − 0.69)	0.018	0.031	0.3 (0.1, 0.86) *p* = 0.026	*p* = 0.028
Number and interval cancer rate according to the time between screening and interval cancer diagnosis							
< 12 months	4 0.24‰ (0.15, 0.35)	7 0.29‰ (0.20, 0.39)	− 0.05 (− 0.37, 0.28)	0.78	0.833	0.84 (0.24, 2.89) *p* = 0.79	*p* = 0.846
≥ 12 months	11 0.68‰ (0.51, 0.8)	33 1.38‰ (1.19, 1.58)	− 0.70 (− 1.32, − 0.08)	0.038	0.065	0.49 (0.24, 0.97) *p* = 0.043	*p* = 0.056
Sensitivity							
Global	86.72% (84.62, 88.82)	77.24% (76.64, 79.84)	9.47% (0.82, 18.13)	0.043	0.080	-	-
Sensitivity according to age groups							
< 60 years	85.71% (83.54, 87.88)	75.72% (73.06, 78.38)	9.98% (− 1.66, 21.64)	0.110	0.177	-	-
≥ 60 years	88.37% (86.38, 90.36)	79.06% (76.54, 81.58)	9.3% (− 3.57, 22.17)	0.193	0.271	-	-

*CBTST*, Córdoba Breast Tomosynthesis Screening Trial; *OR*, odds ratio

95% CI in parentheses

There were no significant differences in interval cancer rate between the two populations when stratified by age group. For those under 60 years of age, the ICR was 0.99‰; 95% CI: 0.73, 1.2 in the study population vs 1.88‰; 95% CI: 1.57, 2.12 in the control population; *p* = 0.160. For those over 60 years of age, the ICR was 0.82‰; 95% CI: 0.52, 1.14 vs 1.71‰; 95% CI: 1.37, 2.05 respectively; *p* = 0.212.

Regarding breast density, the interval cancer rate was also significantly lower in the group of women with dense breasts (categories C and D) (0.95‰; 95% CI: 0.55, 1.35 in the study population vs 3.17‰; 95% CI: 2.64, 3.62 in the control population; *p* = 0.031).

When the rate of interval cancer was analysed according to time between the interval cancer diagnosis and the previous negative screening, no difference was detected between the study and control populations in the first 12 months (0.24‰; 95% CI: 0.15, 0.35 vs 0.29‰; 95% CI: 0.20, 0.39 respectively; *p* = 0.833). However, the differences between the study and control populations tended to be statistically significant after 12 months from the last screening exam (0.68‰; 95% CI: 0.51, 0.8 vs 1.38‰; 95% CI: 1.19, 1.58 respectively; *p* = 0.065). The confidence intervals of the RD and OR support these differences.

### Sensitivity of the screening programme

The overall sensitivity of the screening programme was 80.79% (95% CI: 78.35, 83.23). No significant differences were found between the study population (86.72%; 95% CI: 84.62, 88.82) versus the control population (77.24%; 95% CI: 76.64, 79.84) (*p* = 0.080). In addition, there were no differences when comparing by age group (over and under 60 years of age) (Table 2).

### Characteristics of interval cancers: study population (DBT + DM) vs control population (DM)

The general and pathological characteristics of the interval and screen-detected cancers along with the treatments received by the patients are shown in Tables 3 and 4.

**Table 3:** General, histopathological, and immunohistochemical characteristics of interval and screen-detected cancers

Parameter	Interval cancers Study Group	Interval cancers Control Group	*p* value	Adjusted *p* value	Interval cancers (both groups)	Screening Detected cancers (both groups)	*p* value	Adjusted *p* value
*N* cancers	15	43	-	-	58	244	-	-
Infiltrating	13 (86.7)	42 (97.7)	0.810	0.857	55 (94.8)	183 (75)	< 0.001	< 0.001
In situ	1 (6.7)	0			1 (1.7)	61 (25)		
Missing	1 (6.7)	1 (2.3)	-	-	2 (3.4)	0	-	-
Radiologic involvement								
Mass like	15 (100)	39 (90.7)	0.384	0.394	54 (93.1)	180 (73.8)	< 0.001	< 0.001
Calcifications	0	2 (4.7)			2 (3.4)	64 (26.2)		
Missing	0	2 (4.7)	**-**	-	2 (3.4)	0	**-**	**-**
Size (mm) ^								
Infiltrating	15 ± 2	27 ± 3	0.019	0.031	24 ± 3	18 ± 1	0.014	0.031
DCIS	70	-	-	-	70	27 ± 4	0.109	0.166
Axilla status								
Axilla +	4 (26.7)	17 (39.5)	0.582	0.707	21 (36.2)	48 (19.7)	0.015	0.031
Axilla −	10 (66.7)	24 (55.8)			34 (58.6)	168 (68.9)		
Missing	1 (6.7)	2 (4.7)	-	-	3 (5.2)	28 (11.5)	-	-
Stage								
0–I	6 (40)	10 (23.3)	0.189	0.264	16 (27.6)	170 (69.7)	< 0.001	< 0.001
II–IV	8 (53.3)	31 (72.1)			39 (67.2)	73 (29.9)		
Missing	1 (6.7)	2 (4.7)	-	-	3 (5.2)	1 (0.4)	-	-
Histological degree								
1	3 (20)	3 (7)	0.295	0.391	6 (10.3)	96 (39.4)	< 0.001	< 0.001
2	6 (40)	17 (39.5)			23 (39.7)	90 (36.9)		
3	5 (33.3)	21 (48.8)			26 (44.8)	58 (23.8)		
Missing	1 (6.7)	2 (4.7)	-	-	3 (5.2)	0	-	-
ER								
Positive	10 (66.7)	34 (79.1)	0.452	0.562	44 (75.9)	223 (91.4)	0.001	0.005
Negative	4 (26.7)	8 (18.6)			12 (20.7)	17 (7)		
Missing	1 (6.7)	1 (2.3)	-	-	2 (3.4)	4 (1.6)	-	-
PR								
Positive	9 (60)	29 (67.4)	0.741	0.840	38 (65.5)	196 (80.3)	0.022	0.045
Negative	5 (33.3)	13 (30.2)			18 (31)	44 (18)		
Missing	1 (6.7)	1 (2.3)	-	-	2 (3.4)	4 (1.6)	-	-
HER2								
Positive	2 (13.3)	5 (11.6)	0.742	0.840	7 (12.1)	7 (2.9)	0.015	0.031
Negative	11 (73.3)	37 (86)			48 (82.8)	173 (70.9)		
Missing	2 (13.3)	1 (2.3)	-	-	3 (5.2)	64 (26.2)	-	-
Ki67								
< 20%	6 (40)	18 (41.9)	0.832	0.857	24 (41.4)	137 (56.1)	< 0.001	< 0.001
≥ 20%	8 (53.3)	21 (48.8)			29 (50)	46 (18.9)		
Missing	1 (6.7)	4 (9.3)	-	-	5 (8.6)	61 (25)	-	-

*DCIS*, ductal carcinoma in situ; *ER*, oestrogen receptors; *PR*, progesterone receptors; *HER2*, human epidermal growth factor receptor 2

Unless otherwise indicated, data are number of cancers (percentage)

^Size according to the pathological study of the surgical specimen. Cases of patients treated with neoadjuvant therapy were excluded. Data are shown as mean ± standard deviation

**Table 4:** Treatments in patients with interval and screen-detected cancers

Parameter	Interval cancers study population	Interval cancers control population	*p* value	Adjusted *p* value	Interval cancers (both populations)	Screening detected cancers (both populations)	*p* value	Adjusted *p* value
Breast treatment								
Conserving surgery	7 (46.7)	23 (53.5)	0.213	0.296	30 (51.7)	201 (82.4)	0.1	0.166
Mastectomy	2 (13.3)	8 (18.6)			10 (17.3)	35 (14.3)		
Neoadjuvant	5 (33.3)	11 (25.6)	-	-	16 (27.6)	8 (3.3)	-	-
Missing	1 (6.7)	1 (2.3)	-	-	2 (3.4)	0	-	-
Axilla treatment								
Axillary lymphadenectomy	0	13 (30.2)	0.022	0.045	13 (22.4)	23 (9.4)	0.003	0.013
No axillary lymphadenectomy*	13 (86.7)	29 (67.4)			42 (72.4)	221 (90.6)		
Missing	2 (13.3)	1 (2.3)	-	-	3 (5.2)	0	-	-

^*^Patients with negative and positive sentinel lymph node biopsy (SLNB) meeting ACOSOG Z0011 criteria and patients with no SLNB performed were included

The mean size of the invasive interval cancers, excluding those treated with neoadjuvant therapy, was 15 ± 2 mm in the population studied with DBT + DM and 27 ± 3 mm in the control population screened with DM (*p* = 0.031).

There were no significant differences between the populations regarding the percentage of axillary involvement or the stage of interval cancers. The percentage of patients with axillary involvement in the study population was 26.7% (4/15) compared to 39.5% (17/43) observed in the control population (*p* = 0.707). Regarding the stage of interval cancers detected, 23.3% (10/43) was stage 0–I and 72.1% (31/43) was stage II–IV in the control population, while 40% (6/15) was stage 0–I and 53.3% (8/15) was stage II–IV (*p* = 0.264) in the study population.

No statistically significant differences were detected between interval cancers in the study and control populations in the degree of histological differentiation (*p* = 0.391), percentage of oestrogen receptor positive (ER +) (66.7% vs 79.1%; *p* = 0.562), progesterone receptor positive (PR +) (60% vs 67.4%; *p* = 0.840), human epidermal growth factor receptor 2 positive (HER2 +) (13.3% vs 11.6%; *p* = 0.840), or percentage with Ki67 values > 20% (53.3% vs 48.8%; *p* = 0.857). Regarding surgical treatment, no statistically significant differences were detected in breast surgery (conserving surgery 46.7% in the study population vs 53.5% in the control population and mastectomy in 13.3% and 18.6%, respectively; *p* = 0.296). However, a higher proportion of axillary lymphadenectomy was found in the control population compared to the study population (13/43 (30.2%) vs 0%, respectively; *p* = 0.045).

### Characteristics of interval cancers vs screen-detected cancers

The percentage of infiltrating carcinomas was 94.8% (55/58) in interval cancers compared to 75% (183/244) in screen-detected cancers (*p* < 0.001). The mean size of the invasive component of the tumours, excluding those treated with neoadjuvant therapy, was 24 ± 3 mm in interval cancers and 18 ± 1 mm in screen-detected cancers (*p* = 0.031) (Table 3).

The percentage of patients with axillary involvement was 36.2% (21/58) in interval cancers compared to 19.7% (48/244) in screen-detected cancers (*p* = 0.031). Regarding stages, 69.7% (170/244) of screen-detected cancers were diagnosed in early stages (0–I), while only 27.6% (16/58) of interval cancers were diagnosed in these stages (*p* < 0.001).

Interval cancers showed a lower degree of differentiation and phenotypes associated with a worse prognosis compared to screen-detected cancers, with a higher proportion of ER − (20.7% vs 7%; *p* = 0.005), PR − (31% vs 18%; *p* = 0.045), HER2 + (12.1% vs 2.9%; *p* = 0.031), and Ki67 values ≥ 20% (50% vs 18.9%; *p* < 0.001).

On the other hand, regarding the percentage of women who had conservative surgery, no differences were found between screen-detected cancers and interval cancers (82.4% [201/244] vs 51.7% [30/58], respectively; *p* = 0.166). However, the proportion of axillary lymphadenectomy was lower in patients with screen-detected cancer (9.4% vs 22.4%; *p* = 0.013).

## Discussion

The results of this study show statistically significant differences between the rates of interval cancers in women screened with DBT + DM or DM (0.93‰ vs 1.8‰, respectively; *p* = 0.045), with a lower probability of being diagnosed with an interval cancer in women screened with DBT + DM (OR = 0.51). Likewise, these statistically significant differences are maintained in the subgroup of women with dense breasts (0.95‰ vs 3.17‰; *p* = 0.031) and the results border on statistical significance in those with interval cancers diagnosed at least 12 months after of the previous screening test (0.68‰ vs 1.38‰; *p* = 0.065; RD =  − 0.70; 95% CI: − 1.32, − 0.08).

Although these results differ from most findings published to date, which have not shown significant differences in interval cancer rates between women screened with DBT and DM [13–20], they are similar to those obtained by Johnson K et al [21], who found a lower rate of interval cancer in women screened with DBT. This study did not find differences in the interval cancer rate between women screened with DBT + DM or DM alone when the analysis was stratified according to age group. However, when breast density is considered, differences between both populations were observed in women with dense breasts. To our knowledge, these differences have not been previously analysed in any other study. Moreover, no study has described differences in the rate of interval cancer according to the time between the screening examination and the diagnosis of interval cancer between women screened with DM or DBT. This work’s results have not shown significant differences between women screened with DBT + DM or DM alone in the first 12 months after the previous screening test. However, differences that are almost statistically significant were found after 12 months from the previous test (*p* = 0.065), which may indicate a greater impact of DBT in biennial screenings than in annual ones.

Regarding sensitivity, these data showed a higher sensitivity when screening using DBT + DM (86.72% vs 77.24%), although the differences were not significant (*p* = 0.080). Previous studies have also described a slight improvement in sensitivity with DBT, although the findings did not reach statistical significance either [14, 16, 17, 20].

As for the tumour characteristics of interval cancers in patients screened with DBT + DM or with DM only, most studies did not have enough interval cancer cases for comparison [16, 18] or did not find significant differences [13, 14]. However, the meta-analysis performed by Houssami et al [20] found significant differences in axillary involvement, with a lower proportion of affected axilla in the group screened with DBT. Furthermore, a recent study carried out by Johnson et al [21] indicated that interval cancers in patients screened with DBT tended to be of a smaller size and had a lower proportion of axillary involvement. This work’s data are in line with the findings by Johnson et al: interval cancers in the population screened with DBT + DM (*p* = 0.031) were smaller and there were fewer lymphadenectomies (*p* = 0.045).

Regarding the comparison between interval cancers and screen-detected cancers, the results described herein are consistent with those published by Hovda et al [14] and Bahl et al [18], who found worse prognostic characteristics in interval cancers.

This study has some limitations. First, only patients from a single centre have been included. Second, women from a single round of screening are included where the number of interval cancers is too low to properly compare their characteristics between different techniques. Third, there were four readings for the population screened with DBT + DM compared to two readings for the population screened with DM, which may have led to fewer interval cancers being detected in the group with more readings. Nonetheless, in these series, the double DM reading only led to the detection of six additional cancers in the group screened with DBT, five of which were ductal carcinoma in situ (DCIS), which presumably would not have presented as interval cancers. Finally, it is possible that some interval cancers were missed, despite the comprehensive search carried out.

Screening with DBT + DM led to a lower rate of interval cancer compared to screening with DM alone, especially in women with denser breasts and more than 12 months after the screening test. Interval cancers were smaller in women screened with DBT + DM compared to those screened with DM. Although new studies as well as meta-analyses of published studies are necessary, these results support the benefits of tomosynthesis use in screening programmes.

## Abbreviations

BI-RADS: Breast Imaging Reporting and Data System
CBTST: Córdoba Breast Tomosynthesis Screening Trial
CC: Craniocaudal
CDR: Cancer detection rate
CI: Confidence interval
DBT: Digital breast tomosynthesis
DM: Digital mammography
ER: Oestrogen receptor
HER2: Human epidermal growth factor receptor 2
ICR: Interval cancer rate
MLO: Mediolateral oblique
OR: Odds ratio
PR: Progesterone receptor
RD: Rate difference
SD: Standard deviation
SLNB: Sentinel lymph node biopsy

## Declarations

### Guarantor

The scientific guarantor of this publication is Marina Álvarez Benito.

### Statistics and biometry

One of the authors has significant statistical expertise: Pilar Font Ugalde.

• Maimónides Biomedical Research Institute of Córdoba (IMIBIC) Córdoba, Spain.

• University of Córdoba, Spain.

• Reumatology Service, Reina Sofía University Hospital, Menéndez Pidal Avenue s/n, 14004 Córdoba, Spain.

María Pata (Biostatech) kindly provided statistical advice for this manuscript.

### Study subjects or cohorts overlap

Some study subjects or cohorts (study population) have been previously reported in:

-*Romero Martín S, Raya Povedano JL, Cara García M, Santos Romero AL, Pedrosa Garriguet M, Álvarez Benito M (2018) Prospective study aiming to compare 2D mammography and tomosynthesis* + *synthesized mammography in terms of cancer detection and recall. From double reading of 2D mammography to single reading of tomosynthesis. Eur Radiol 28(6):2484–91*.

This paired trial compared the screening performance of DM versus DBT in terms of recall and detection rate.

-*Jose Luis Raya-Povedano MD, Sara Romero-Martín phD MD, Esperanza Elías-Cabot MD, Albert Guberm-Mérida, Alejandro Rodriguez-Ruiz phD and Marina Álvarez-Benito phD MD (2021) AI-based strategies to reduce workload in breast cancer screening with mammography and tomosynthesis. Radiology 000:1–9.*

This publication studied the ability of artificial intelligence to reduce the workload of reading screening.

*- Sara Romero-Martín phD MD, Esperanza Elías-Cabot MD, Jose Luis Raya-Povedano MD, Albert Guberm-Mérida, Alejandro Rodriguez-Ruiz phD and Marina Álvarez-Benito phD MD (2022) Stand-alone use of artificial intelligence form digital mammography and digital breast tomosynthesis screening. Radiology 302: 535–542.*

This paper retrospectively evaluated the stand-alone performance of an artificial intelligence systems as an independent reader of DM and DBT examinations.

### Methodology

• retrospective

• diagnostic or prognostic study

• performed at one institution
